# The Role of Hemoglobin Subunit Delta in the Immunopathy of Multiple Sclerosis: Mitochondria Matters

**DOI:** 10.3389/fimmu.2021.709173

**Published:** 2021-08-24

**Authors:** Afshin Derakhshani, Hossein Safarpour, Mahdi Abdoli Shadbad, Nima Hemmat, Patrizia Leone, Zahra Asadzadeh, Mehrdad Pashazadeh, Behzad Baradaran, Vito Racanelli

**Affiliations:** ^1^Immunology Research Center, Tabriz University of Medical Sciences, Tabriz, Iran; ^2^Laboratory of Experimental Pharmacology, Istituto di Ricovero e Cura a Carattere Scientifico (IRCCS) Istituto Tumori Giovanni Paolo II, Bari, Italy; ^3^Cellular and Molecular Research Center, Birjand University of Medical Sciences, Birjand, Iran; ^4^Student Research Committee, Tabriz University of Medical Sciences, Tabriz, Iran; ^5^Department of Biomedical Sciences and Human Oncology, University of Bari “Aldo Moro”, Bari, Italy; ^6^Department of Immunology, Faculty of Medicine, Tabriz University of Medical Sciences, Tabriz, Iran

**Keywords:** multiple sclerosis, mitochondrial injury, oxidative stress, immune cells, peripheral blood mononuclear cells, HBD, single-cell RNA sequencing

## Abstract

**Background:**

Although the exact pathophysiology of MS has not been identified, mitochondrial stress can be one of the culprits in MS development. Herein, we have applied microarray analysis, single-cell sequencing analysis, and *ex vivo* study to elucidate the role of mitochondrial stress in PBMCs of MS patients.

**Methods:**

For this purpose, we analyzed the GSE21942 and GSE138266 datasets to identify the DEGs and hub genes in the PBMCS of MS patients and describe the expression of shared genes in the different immune cells. The GO pathway analysis of DEGs and turquoise module genes were conducted to shed light on their biological significance. To validate the obtained results, the gene expression of *HBD*, as the most remarkable DEG in the PBMCS of affected patients, was measured in the PBMCS of healthy donors, treatment-naïve MS patients, and MS patients treated with GA, fingolimod, DMF, and IFNβ-1α.

**Results:**

Based on WGCNA and DEGs analysis, *HBD, HBM, SLC4A1, LILRA5, SLC25A37, SELENBP1, ALYREF, SNRNP40*, and *HINT3* are the identified common genes in the PMBCS. Using single-cell sequencing analysis on PBMCS, we have characterized various cell populations in MS and illustrated the common gene expression on the different immune cells. Furthermore, GO pathway analysis of DEGs, and turquoise module genes have indicated that these genes are involved in immune responses, myeloid cell activation, leukocyte activation, oxygen carrier activity, and replication fork processing bicarbonate transport pathways. Our *ex vivo* investigation has shown that *HBD* expression in the treatment-naïve RRMS patients is significantly increased compared to healthy donors. Of interest, immunomodulatory therapies with fingolimod, DMF, and IFNβ-1α have significantly decreased *HBD* expression.

**Conclusion:**

*HBD* is one of the remarkably up-regulated genes in the PBMCS of MS patients. *HBD* is substantially up-regulated in treatment-naïve MS patients, and immunomodulatory therapies with fingolimod, DMF, and IFNβ-1α can remarkably down-regulate *HBD* expression. Based on the currently available evidence, the cytoprotective nature of HBD against oxidative stress can be the underlying reason for HBD up-regulation in MS. Nevertheless, further investigations are needed to shed light on the molecular mechanisms of HBD in the oxidative stress of MS patients.

## Introduction

MS is an inflammatory demyelinating disease of the CNS that affects more than 2.5 million people worldwide (1). In MS, myelin-directed immunity led by immune cells’ infiltration to CNS can damage the myelin sheath of axons, oligodendrocytes, and neurons (2).

Since epidemiological studies have shown that the relatives of affected individuals are at a higher risk of developing severe MS, genetic factors have been implicated in its pathogenesis (3, 4). However, environmental factors, e.g., latitude, also have roles in its pathogenesis (5). Therefore, it is commonly considered as the result of multifactorial factors, i.e., genetic predisposition and exposure to certain environmental factors. Acute inflammation, which leads to chronic inflammation, is the cornerstone of MS initiation. The release of pro-inflammatory cytokines and ROS have been implicated in MS progression (6). Growing evidence has indicated that mitochondrial oxidative stress plays a pivotal role in the pathogenesis of MS (7). Witte et al. have shown that mtHSP70, as the biomarker of mitochondrial stress, has been remarkably up-regulated in MS lesions (8). Furthermore, Gonzalo et al. have demonstrated that the redox status of PBMCs has been considerably impaired, leading to ROS overproduction (9). In line with these, the results of randomized clinical trials have shown that administrating coenzyme Q10, as a potent antioxidant, can lead to promising results for MS patients (10–12). Therefore, a better understanding of the mitochondrial stress in PBMCs is needed for developing novel targeted therapy for MS patients.

Technological, proteomics, and metabolomics offer ample opportunities to unravel molecular mechanisms involved in MS pathogenesis. WGCNA, one of the systems computational approaches, is an easy way to correlate genes with similar expression patterns (13–15). It also can be extended to uncover highly correlated molecules and separate group modules, revealing the connection between hub genes and external sample traits. On the other hand, scRNA-seq has facilitated novel and deeper insight into the expression of marker genes. Therefore, we have used microarray and scRNA-seq data to identify essential genes involved in MS pathogenesis and investigate their interactions as a unique system. In addition, we have investigated the expression of *HBD*, as a gene that has critical roles in oxidative stress, in healthy donors, treatment-naïve MS patients, and MS patients treated with GA, fingolimod, DMF, and IFNβ-1α to validate the results of *in-silico* analysis.

## Materials and Methods

### *In-Silico* Analysis

#### Microarray Data Study

The GSE21942 microarray dataset was downloaded from the GEO database (https://www.ncbi.nlm.nih.gov/geo/). This dataset was based on the Agilent GPL570 platform (HG-U133_Plus_2 Affymetrix Human Genome U133 Plus 2.0 Array) and included 29 samples, i.e., PBMCs from MS patients and healthy subjects (16). The raw data were corrected, quantile-normalized, and probe IDs were converted to gene symbols. Gene symbols were filtered across all samples through their variance. Only genes with variances ranked in the top 5000 were selected for subsequent analyses.

##### Identification of DEGs

The R software was used to identify the DEGs between the PBMCs of healthy individuals and MS patients. After analyzing values in each sample, adjusted p-value<0.0001 and |logFC|≥2 were set as the cut-off criteria. Besides, the up-and down-regulated genes, -log (adjusted p-value), and logFC of each gene were used to plot the volcano plot.

##### Constructing Co-Expression Modules in Multiple Sclerosis

A co-expression network for the gene expression data related to patient and healthy groups has been reconstructed using the protocols of the WGCNA package. Following the scale-free topological algorithm, when the β value was set to 8, the adjacency matrix met the scale-free topology criteria. Based on the adjacency matrix, the TOM and dis-TOM were achieved. Finally, as clusters of highly interconnected genes, the modules were defined with a minimum module size of 30 genes per module and a cut height of 0.004.

##### Constructing Module-Trait Relationships in Multiple Sclerosis

To recognize modules that were significantly related to the evaluated clinical trait, the expression profiles of each module were summarized by its ME as the eigenvector correlated to the first principal component of the expression matrix. The GS values were used for measuring individual genes’ associations with disease. Also, MM was defined as the correlation of the ME and the gene expression profile for each module. If the GS and MM were highly correlated, the most significant (central) elements in the modules were also closely associated with the trait. So, they could be used to construct the network and identify the hub-genes. Finally, genes with both GS and MM ≥0.86 were chosen as hub genes. Furthermore, a co-expression network consisted of hub genes was constructed by geneMANIA plug-in Cytoscape v3.8.1 (17).

##### Identification of Common Hub Genes and DEGs

Venn diagram was generated using the “Venny” v 2.1 software freely available (http://bioinfogp.cnb.csic.es/tools/venny/) to identify common genes between hub genes and DEGs. Common genes were considered the central genes correlated to MS pathogenesis and applied for further analyses.

##### Heatmap Analysis for Common Genes

In the current study, we used heatmap analysis to demonstrate the visualized differences of common hub genes and DEGs between the PBMCs from healthy individuals and MS patients.

##### Functional Annotation of the MS Correlated Module Genes and DEGs

To reveal the biologic function and pathway of selected modules and DEGs, functional GO terms and KEGG pathway were enriched using ClueGO v2.5.7 and CluePedia v1.5.7 plug-in of Cytoscape v3.8.1 (17). Enriched ontological terms and pathways were conducted with the threshold of Benjamin-adjusted *p*-value< 0.001.

##### PPI Network

The identified turquoise module members and hubs were subjected to STRING v11 plug-in of Cytoscape v3.8.1 to find possible PPI with the confidence score ≥ 0.700 and 0 interactors as the cut-off criteria (17, 18). The predicted PPI networks were then analyzed with the MDOCE v1.5.1 to detect highly interconnected regions (clusters) with the following cut-off criteria, node degree ≥ 2, node score ≥ 0.2, node density ≥ 0.5, and without haircutting (19).

#### Single-Cell Transcriptome Analysis of Common Candidate Genes in MS Samples

##### Data Acquisition, Quality Control, and Dimensionality Reduction

As we shown in the previous study,we assessed the raw data from a study by Schafflick et al. (20, 21). The original survey applied single-cell transcriptomics to PBMC and CSF cells from MS patients and controls and validated vital findings. The raw single-cell RNA-seq data from their study were deposited in GEO (GSE138266) (22). The Scanpy toolkit was leveraged for data analyses (23). First, quality control was performed to filter low-quality cells. For this purpose, we only retained cells that had (1) more than 500 genes, (2) less than 17500 counts, and (3) less than 20% of reads mapped to mitochondrial genes. Normalized expression is calculated using the *normalize_total* function in Scanpy, or the calculates factors from SCRAN, which could estimate size factors for each cell to remove bias within the cell counts and improve cross-cell comparison of cell expression values. To enable unsupervised clustering and cell-type identification, dimensionality reduction was performed with the top 4000 most highly variable genes for PCA. PCA on the combined set of samples for each sample after selection of highly variable genes. Once embedded in this PCA space, we constructed a nearest neighbor graph identifying the k = 15 nearest neighbors for each cell. We derived uniform manifold approximation (UMAP) embeddings presented for visualization from this most relative neighbor graph using a minimum distance of 0.5 and a spread of 1.0 (24).

##### Clustering and Cell-Type Identification

We used Louvain community detection to the nearest neighbor graph constructed in PCA space to define a cluster partition (21, 25). To annotate the clusters, we used two marker sets. First, differential expression test was performed by a Welch t-test with overestimated variance to find genes that are up-regulated in the cluster compared to all other clusters (marker genes). Second, using official CellMarker website (http://biocc.hrbmu.edu.cn/CellMarker/), we found marker genes of each cell type. This database comprised 2867 cell type marker sets and 467 cell types from 1764 studies.

### *Ex Vivo* Validation

#### Patients and Samples

Forty-five MS patients and twenty-four healthy donors were enrolled in this study. The study was approved by the Ethics Committee of Tabriz University of Medical Sciences (IR.TBZMED.REC.1399.074), and all participants received written informed consent. Demographic data were collected by a questionnaire. Participants with other comorbidities were excluded from the study.

#### Sample Collection, RNA Extraction, and cDNA Synthesis

Ten milliliters (ml) of venous blood were collected to isolate PBMC by the Ficoll method. Total RNA was isolated using TRIzol, according to the manufacturer’s instructions (Riboex, Gene All Biotechnology, Seoul, Korea). Then, cDNA synthesis was performed using a cDNA Reverse Transcription kit (BioFact, South Korea).

#### Real-Time PCR

Relative expression of the *HBD* gene was measured by real-time PCR. 2X Master Mix with high ROX (Biofact, Korea) was used for the current study. The real-time PCR conditions were as follows: initial denaturation 13 min, 95°C, 45 cycles of denaturation; 13s, 95°C; annealing, 30s, 60°C; elongation 20s, 72°C. The specific primer sequences which were used in this study are mentioned as follows: HBD (F:5′‐TCACGGCTGAGATTCGACAG‐3′, R:5′‐ CCTGCGAGAGCCATAGCATC‐3’) and GAPDH (F:5′‐AAGGTGAAGGTCGGAGTCAAC‐3′, R: 5′‐GGGGTCATTGATGGCAACAA‐3′). GAPDH was used as an internal control. Relative gene expression was calculated using the comparative 2^-delta CT^ method.

#### Statistical Analysis

The R software (version 4.0.2) and GraphPad Prism (version 7.05) (GraphPad Software, Inc., San Diego, CA) were used to conduct the statistical analysis. The utilized R packages in this study were WGCNA, Biobase, GEOquery, LIMMA, AgiMicroRna, Affy, pheatmap, reshape, and Rmisc as ggplot2, which could be used to plot the analyzed data. Differences between the study groups were tested *via* one-way analysis of variance (ANOVA). The data were presented as mean ± SD, and p < 0.05 was considered significant for all tests.

## Results

### *In-Silico* Analysis

#### Microarray Data Study

##### Identification of DEGs

**Figure 1:**
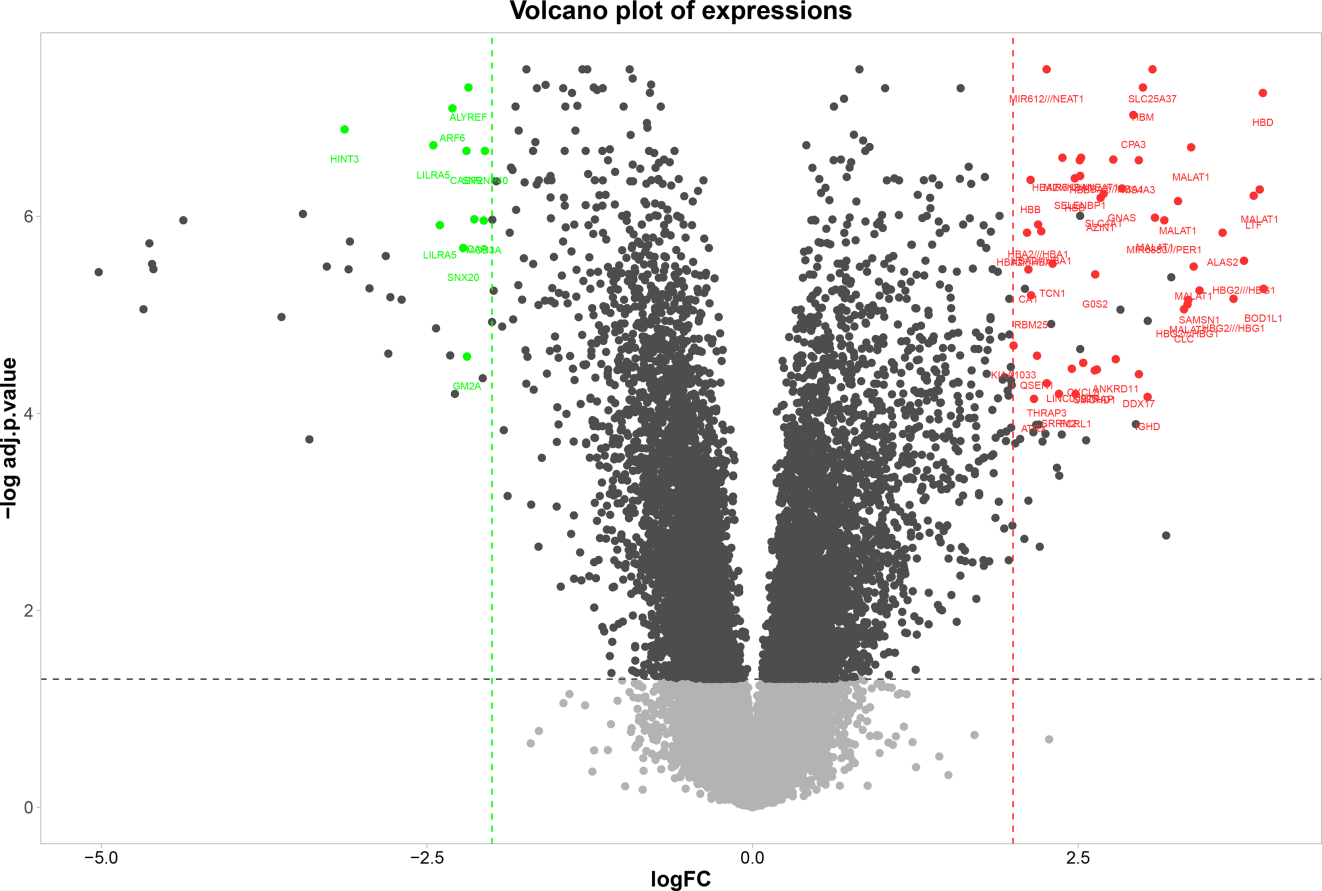
Volcano plot of DEGs between MS and healthy samples. Volcano plot, the vertical axis (y-axis) is the mean value of- log 10 (adj P-value), and the horizontal axis (x-axis) is the value of logFC. Red dots denote the up-regulated genes; green dots represent the down-regulated genes.

**Figure 2:**
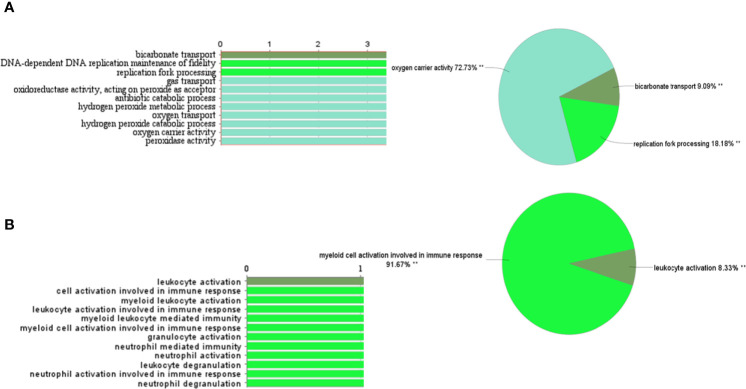
Gene ontology and pathway analysis of DEGs and turquoise module genes through CluePedia. **(A)** The most important biological functions and pathways of the candidate DEGs. **(B)** GO and pathway related to turquoise genes.

A total of 62 genes have been identified as DEGs with the threshold of adjusted p-value< 0.0001 and |logFC|≥2, including 51 up-regulated and 11 down-regulated genes in the PBMCs of MS patients compared to the PBMCs of healthy individuals (**Figure 1**). The most up-regulated genes are *SLC25A37*, *HBD, NEAT1*, and the most down-regulated ones were *ALYREF*, *ARF6*, and *HINT3*. These 62 DEGs were then selected for subsequent analyses. The most important biological functions and pathways of the candidate DEGs have been oxygen carrier activity, replication fork processing, and bicarbonate transport (**Figure 2A**).

##### The Identification of Weighted Gene Co-Expression Network Analysis Module

Based on the variance of expression values, a total of 5000 genes have been included in WGCNA. Two outliers have been observed; thus, the rest of the samples have been included for further assessment (**Figure S1**). Afterward, β=8 has been selected as soft-threshold power, and the weighted co-expression network of MS patients and normal samples have been reconstructed (**Figure S2**). Then, a hierarchical clustering dendrogram has identified modules and illustrated them in branches of the dendrogram with different colors (**Figure S3**). The number of genes on each module varied from 42 (darkolivegreen) to 898 (turquoise) (**Table S1**). Also, 77 genes have not been classified into any modules (designated as grey).

##### Module-Trait Association Analysis

Eigengenes have been calculated for each module to determine the association of the modules with the presence of disease in samples and module-module correlation. It has been indicated that the turquoise module can be positively correlated with multiple sclerosis (r= 0.93, *p*-value=2.00E-12) (**Figure S4**, and **Table S1**). The turquoise module genes enrichment that they were involved in myeloid cell activation during immune responses and leukocyte activation (**Figure 2B**).

##### Hub-Genes Detection and Enrichment Analysis

**Figure 3:**
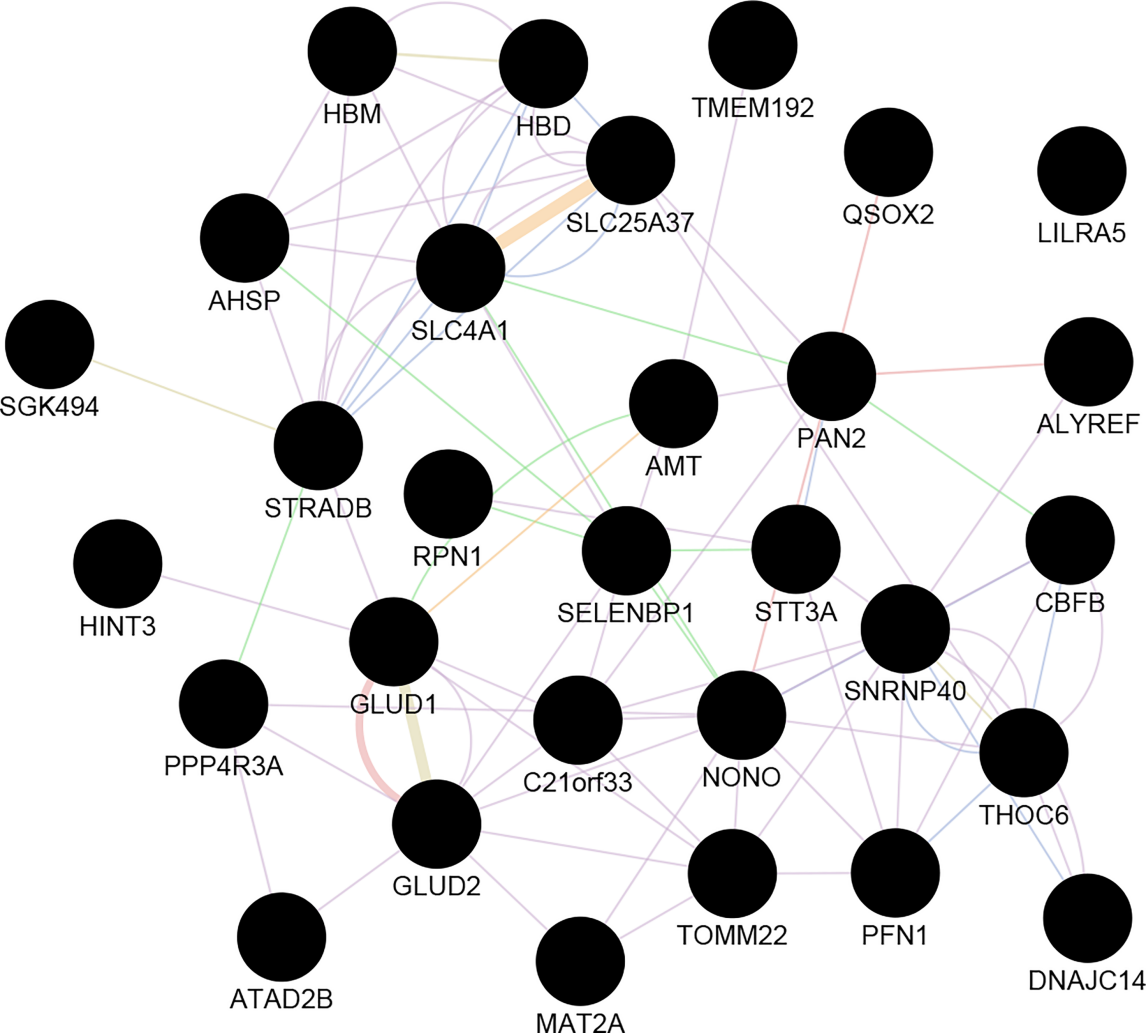
The co-expression network of Hub-genes. Hub genes imported to GeneMANIA to construct a co-expression and gene interactions network. The circle is representing the genes. Edge: lines represent interactions between two genes (multiple lines correspond to multiple sources).

The correlation between features (MM and GS) of the turquoise module can detect the hub-genes of interest that are highly associated with MS pathogenesis (**Figure S5**). The co-expression network of hub genes has been reconstructed using GeneMANIA and Cytoscape software. These Hub-genes are *SELENBP1*, *HBD, HBM, AHSP, HINT3, SNRNP40, DNAJC14, STRADB, STT3A, ALYREF, SLC25A37*, *ILRA5, MAT2A, GLUD2, ATAD2B, GLUD1, TOMM22*, etc. (**Figure 3**).

##### Common Hub Genes and DEGs

**Figure 4:**
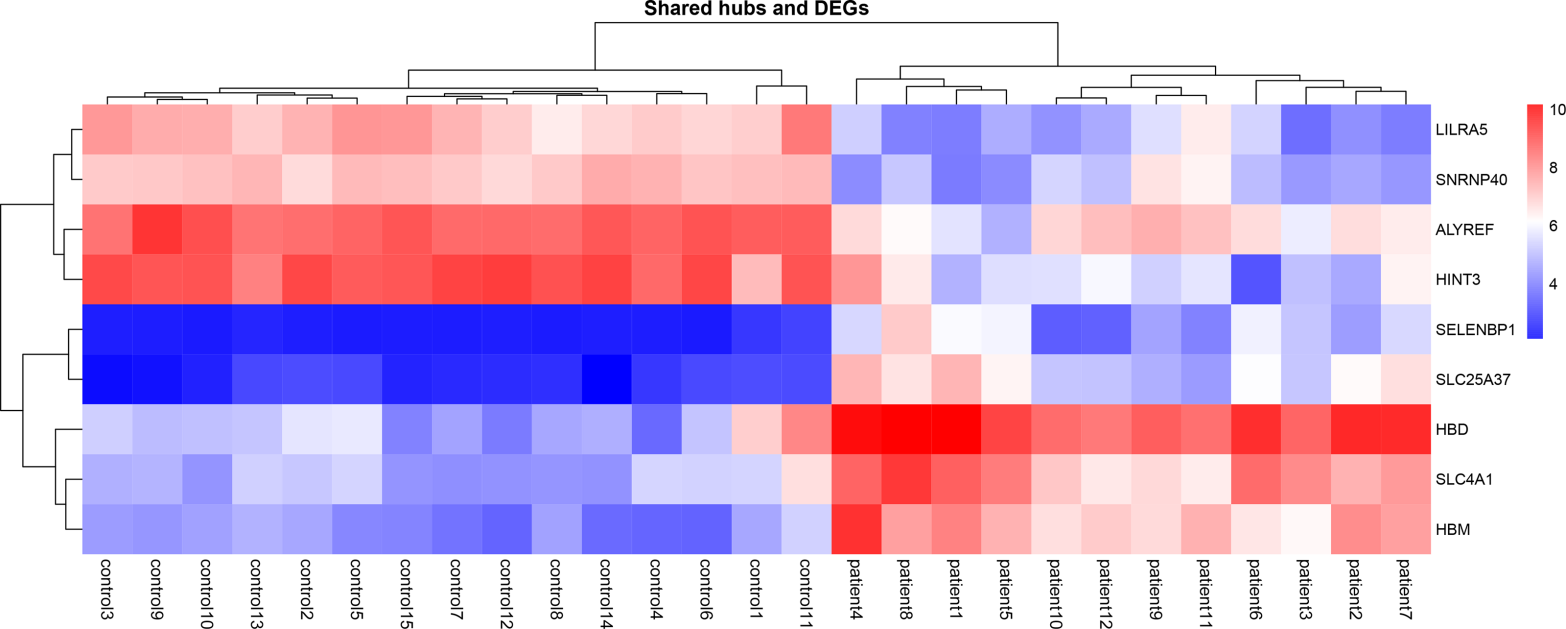
Heatmaps of common gene expression related to the MS and healthy samples. The up-regulated and down-regulated genes are shown as a red and blue pallet, respectively.

Nine genes, i.e., *HBD*, *HBM*, *SLC4A1*, *LILRA5*, *SLC25A37*, *SELENBP1, ALYREF, SNRNP40*, and *HINT3*, have been defined as the primary common genes between the turquoise module and DEGs for further assessment (**Table 1**). The average logFc of candidate genes has varied from -2.20 to 3.9. Besides, the expression value of the hub genes in patients and healthy individuals of selected datasets is illustrated in the heatmap (**Figure 4**).

**Table 1:** The log FC of common candidate genes.

Gene symbol	Log FC	Adj P-Value	Average Log FC	Up-/Down regulation
**HBD**	3.917962	5.57E-08	3.917962	Up-regulated
**SELENBP1**	2.514832	3.88E-07	2.514832	
**HBM**	2.997071	4.90E-08	2.997071	
**SLC4A1**	0.153719	0.295992	1.424233	
**SLC4A1**	2.694747	5.89E-07		
**SLC25A37**	0.063121	0.585563	0.766813	
**SLC25A37**	0.112888	0.74432		
**SLC25A37**	0.460471	0.002461		
**SLC25A37**	0.658764	0.001326		
**SLC25A37**	0.713774	0.00056		
**SLC25A37**	1.173177	5.68E-06		
**SLC25A37**	1.210109	8.29E-05		
**SLC25A37**	1.249593	8.54E-06		
**SLC25A37**	1.25942	3.90E-06		
**SLC25A37**	3.070904	3.21E-08		
**LILRA5**	-2.45055	1.89E-07	-1.1595975	Down-regulated
**LILRA5**	-2.40091	1.23E-06		
**LILRA5**	0.046202	0.410648		
**LILRA5**	0.166868	0.292597		
**ALYREF**	-2.18091	4.90E-08	-1.6205	
**ALYREF**	-1.06009	7.72E-06		
**SNRNP40**	-2.05482	2.16E-07	-2.05482	
**HINT3**	-3.13221	1.31E-07	-2.2038933	
**HINT3**	-1.79537	1.34E-07		
**HINT3**	-1.6841	3.04E-06		

Shared genes between the turquoise module and DEGs (Up/down-regulated genes). A p-value less than 0.05 is statistically significant.

##### The Co-Expression of Hubs and PPI Network of Turquoise Module Members

**Figure 5:**
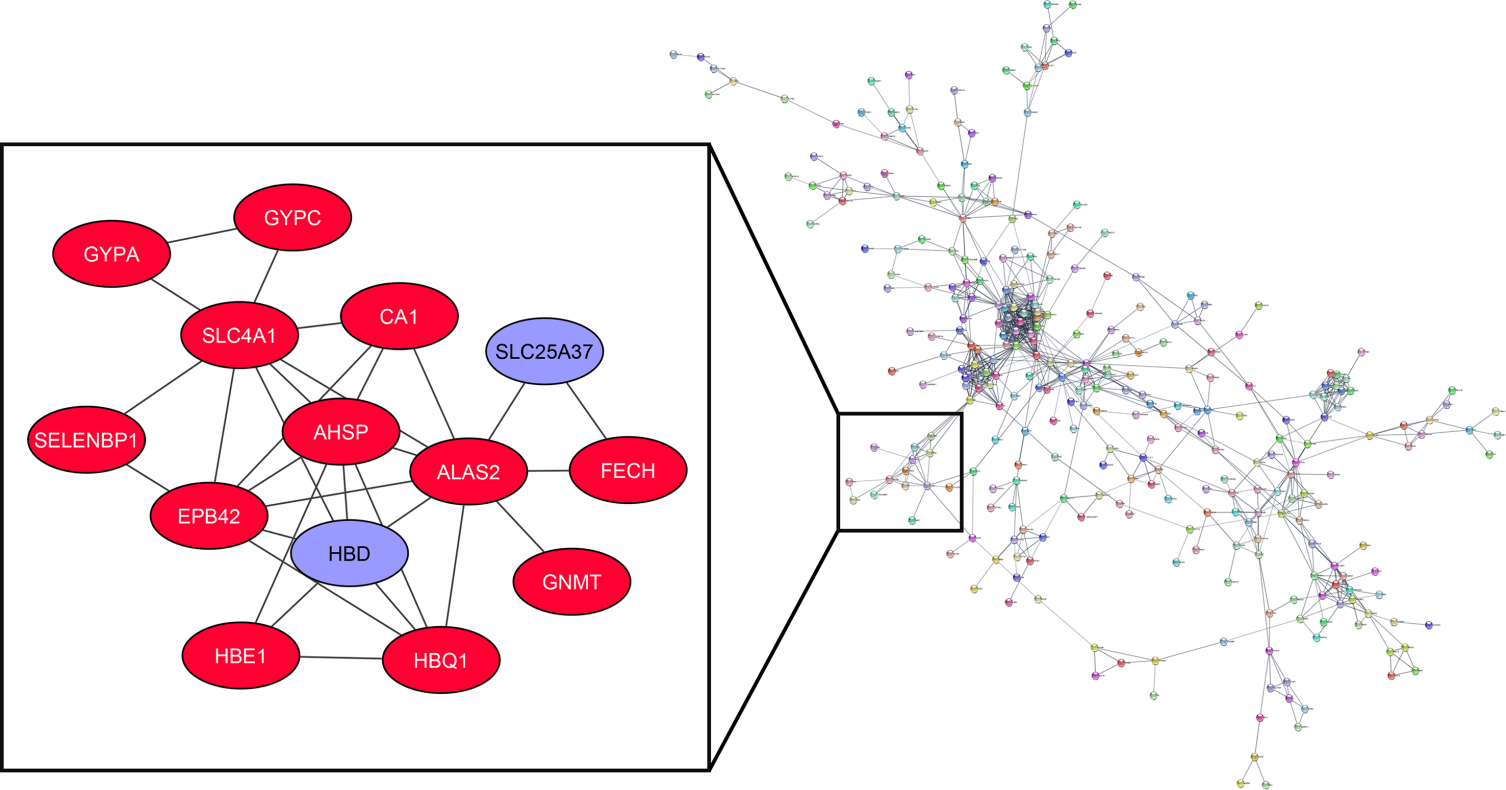
Co-expression of hubs and PPI network of turquoise module members.

The association of turquoise members at the protein level has been analyzed by the STRING plug-in of Cytoscape. Among the 898 genes related to the mentioned module, 311 are in the same network according to the cut-off criteria (**Figure 5**). *HBD* and *SLC25A37*, as highly expressed genes in the MS group based on the DEG analysis, are considered in a cluster network with MCODE score=4.615. Our results have shown that *HBD* can interact with *AHSP*, *HBE1*, *HBQ1*, *ALAS2*, *EPB42*, and *SLC4A1*. Besides, *SLC25A37* can interact with *FECH* and *ALAS2*.

#### Single-Cell Transcriptome Analysis of Common Candidate Genes in MS Samples

##### Differential Cell-Type Proportion Analysis

**Figure 6:**
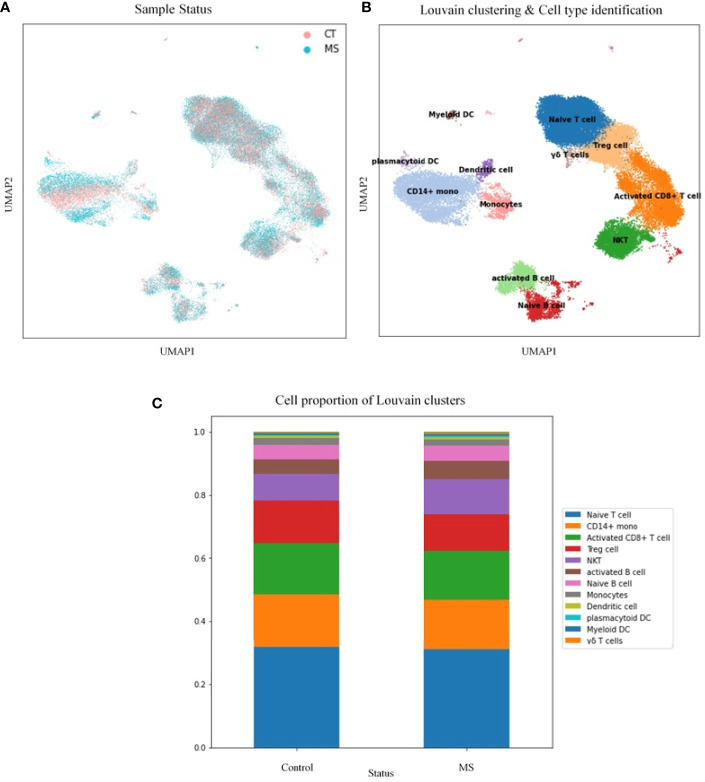
Transcriptomic comparison of MS *versus* control PBMCs. **(A)** UMAP projection of cells with the normal situation (n=17138) colored in dark blue and cells from MS samples (n=25831) were visualized in light blue. **(B)** Louvain clustering and cell annotation was employed to identify 12 major cell populations. **(C)** Bar graphs quantify and compare the proportion of single-cell data of control and MS samples.

Next, we have performed single-cell transcriptome analyses to characterize cell-type-specific molecular signatures of MS in all sub-type of PBMCs. Schafflick et al. recently reported the molecular signature of MS pathogenicity in CSF and PBMCs samples using scRNA-seq technologies (20). Since most of the cells in this dataset are PBMC cells, this dataset can provide valuable data for understanding the expression profile of our hub genes. We use the latest development package, Scanpy, to analyze the scRNA-seq data (23). A total of 40515 single-cell transcriptomes have passed stringent quality control measures. Louvain clustering and cell annotation have been employed to identify significant cell populations. We have assessed the distribution of cell numbers for each cluster by comparing the total number of cells from the MS group to controls. As shown in **Figure 6**, 12 different cell types have been clustered based on the specific markers between control and MS patients. Based on marker genes from scanpy (**Figure S6**) and CellMarker database, we annotated these clusters. The final marker genes is listed in **Table 2**. Cell types were including Naive T cell, CD14+ mono, Activated CD8+ T cell, Treg cell, NKT, Activated B cell, Naive B cell, Monocytes, Dendritic cell (DC), Plasmacytoid DC, Myeloid DC, and γδ T cells. We compared the proportion of the classified PBMC cells and found that healthy derived cells contain more Treg cell but contain fewer NKT and Activated B cell. (**Figure 6**). However these differences between cell types were not significant.

**Table 2:** Marker genes used for cell type annotation in the current study.

Cell type	Marker gene
**Treg cell**	*CCR10, CCR4, IL2RA, FOXP3, CTLA4*
**Naive B cell**	*CD74, CD79A, CD37, IGHD*
**Activated B cell**	*CD74, CD79A, CD27, IGHM*
**Naive T cell**	*CCR7, TCF7, NOSIP, LEF1*
**Monocytes**	*FCGR3A, MS4A7*
**γδ T cells**	*TRDC*
**NKT**	*NKG7, GNLY, KLRC1, FLT3*
**Dendritic cell**	*CD1C, FCER1A, CLEC10A*
**CD14+ mono**	*CD14, FCGR3A*
**Activated CD8+ T cell**	*CD8A, CD8B, CCL5*
**Myeloid DC**	*LYZ, BATF3*
**Plasmacytoid DC**	*TCF4, TNFRSF21*

##### Visualisation of Shared Genes in a Single Cell Resolution

**Figure 7:**
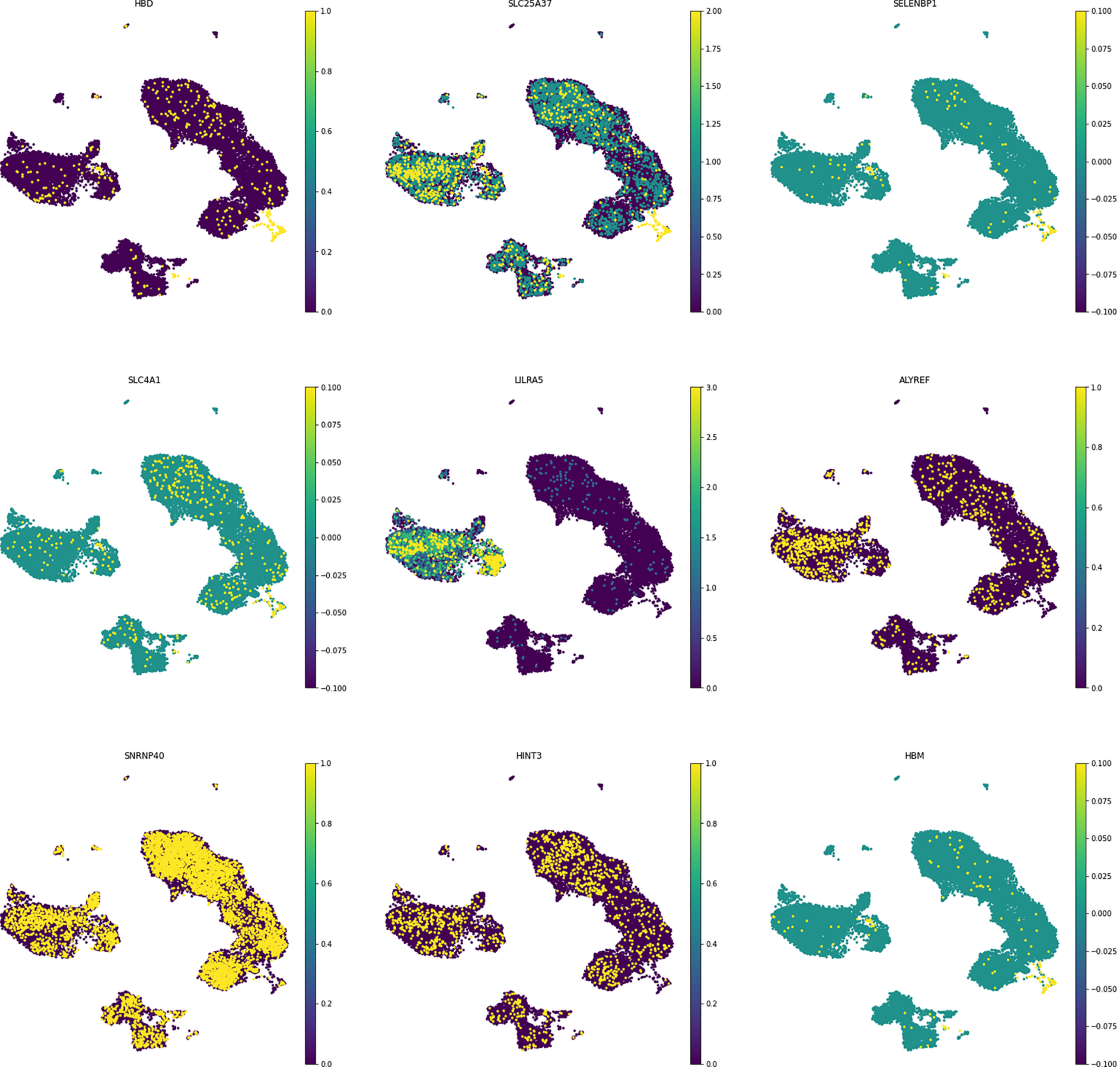
Selected hub-genes expression in different cell populations. The shared genes showed different expression values between other immune cells.

**Figure 8:**
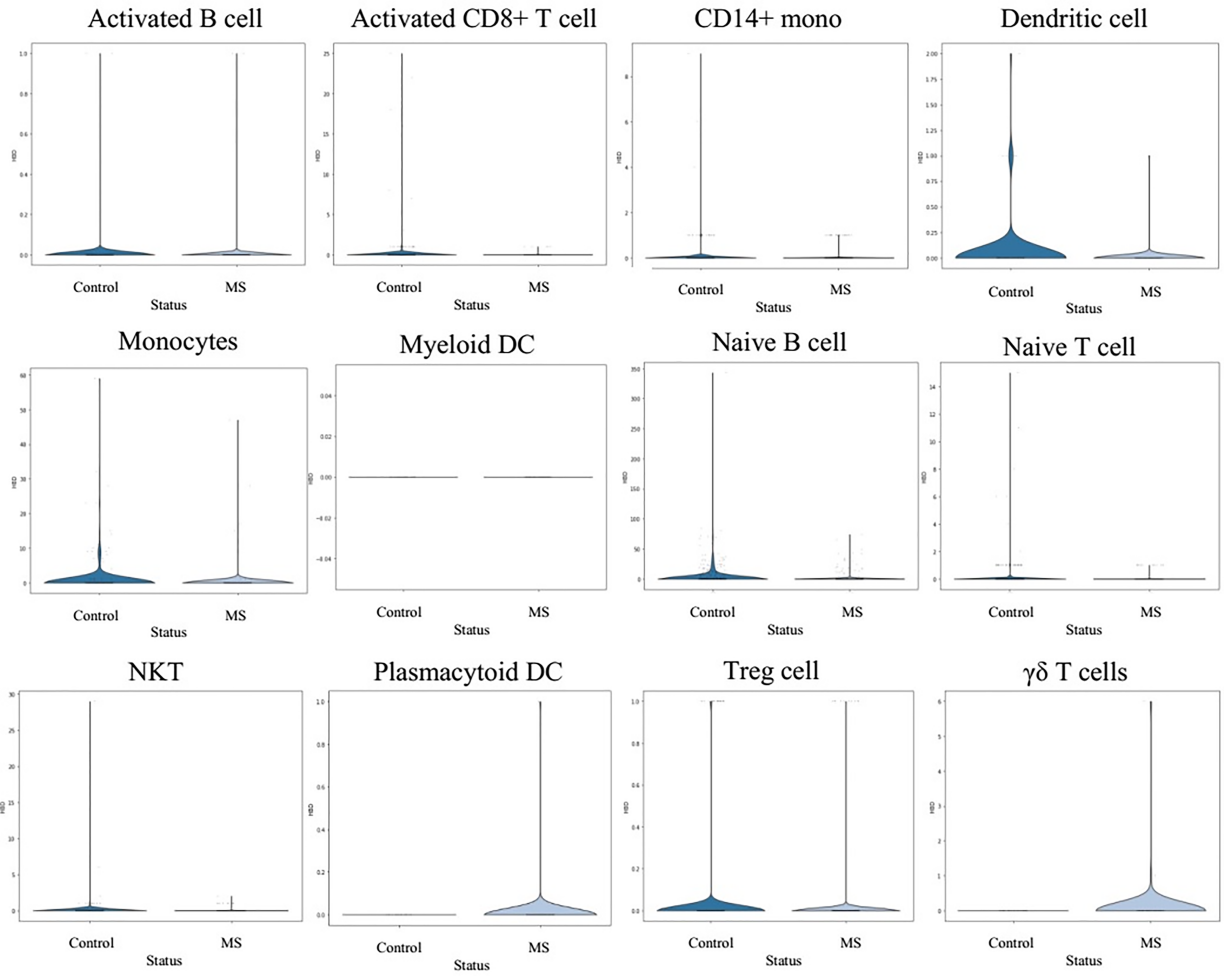
Expression behaviour of *HBD* gene in different Louvain cluster.

In order to understand the gene expression behaviour of shared hub-genes from WGCNA part at a single cell resoloution, the expression pattern of different PBMC cells were visualized using UMAP (**Figure 7**). As depicted in **Figure 7**, *HBD, SELENBP1, HBM, SLC4A1, SLC25A37, LILRA5, ALYREF, SNRNP40*, and *HINT3* expression behaviour were compared in different louvain clusters. Among them, only *LILRA5* was expressed in specific cell types (Monocytes and CD14+ mono cells), and other genes were expressed in all clusters. Based on consideration of *HBD* gene for downstream analysis, we compared expression of this gene in different clusters between control and MS samples. Interestingly, the result showed elevated expression values of *HBD* gene in Plasmacytoid DC and γδ T cells in MS samples while DC, Monocytes, Naive B cell, and Treg cells showed a decreased expression in MS samples. Other cell populations didn’t show significant differences for HBD expression between control and MS samples (**Figure 8**).

### *Ex Vivo* Study

In the current study, we have enrolled forty MS patients who received fingolimod, DMF, IFNβ-1α, and GA. Five MS patients, who have not received any agents, have been considered treatment-naïve patients. The demographical and clinical features of the patients and healthy donors are demonstrated in **Table 3**.

**Table 3:** Classification based on the sex and medication.

Groups	Fingolimod (n=10)	IFNβ-1α (n=10)	DMF (n=10)	GA (n=10)	Naïve patients(n=5)	Healthy control(n=24)
**Age (Mean age ± SD)**	34.3 ± 6.1	35.1 ± 10.3	28 ± 6	33.7 ± 7.2	34 ± 5	29.54 ± 7.4
**Female n (%)**	7 (70%)	7 (70%)	7 (70%)	7 (70%)	4 (80%)	14 (58.3%)

IFNb-1a, Interferon-beta 1-alpha; GA, Glatiramer acetate; DMF, Dimethyl fumarate, SD, standard deviation.

#### The Expression Levels of the *HBD* in the PBMCs of MS Patients and Healthy Individuals

**Figure 9:**
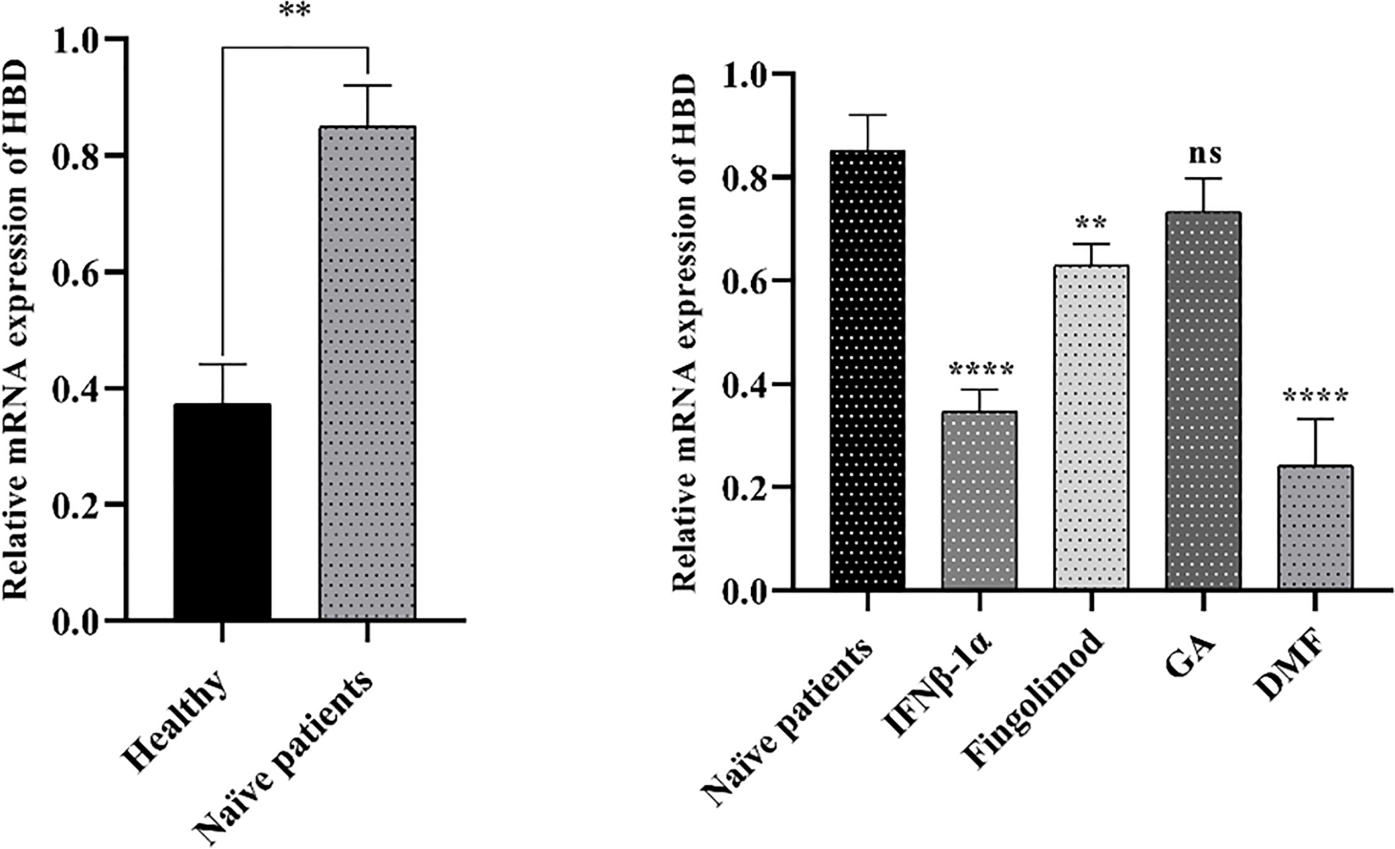
The expression levels of the *HBD* in the MS patients and healthy groups. *HBD* mRNA expression was significantly up-regulated in the treatment-naïve MS patients compared with healthy individuals (left chart). Besides, the expression of *HBD* in MS patients treated with fingolimod, DMF, and IFNβ-1α showed a significant decrease compared to the treatment-naïve MS patients (right chart). GAPDH was used as a housekeeping gene; data are expressed as the mean of 2^−Δct^ (± SD). **P-value< 0.01, ****P-value< 0.0001, and ns, not significant.

The real-time PCR technique has been used to evaluate the gene expression of *HBD* in the PBMCs of MS patients treated with fingolimod (n=10), DMF (n=10), IFNβ-1α (n=10), and GA (n=10), and treatment-naïve MS patients (n=5). As shown in **Figure 9**, *HBD* mRNA expression has been significantly up-regulated in the treatment-naïve MS patients compared with healthy individuals (P < 0.01). Besides, the *HBD* expression levels in MS patients treated with fingolimod, DMF, and IFNβ-1α have been significantly decreased compared to the treatment-naïve MS patients (P<0.01, P<0.0001, and P<0.0001, respectively) (**Figure 9**).

## Discussion

It takes gathering pieces of information over time to comprehend MS pathogenicity (26, 27). Critical information that should be integrated includes the genetic background and the cross-talk between the immune system and CNS (28). To accomplish this aim, we have applied a microarray dataset related to PBMCs from MS patients and healthy individuals to evaluate the gene expression using the WGCNA package. We have investigated the common hub genes and DEGs below: *HBD, SELENBP1, HBM, SLC4A1, SLC25A37, LILRA5, ALYREF, SNRNP40*, and *HINT3.* Moreover, GO pathway analysis of DEGs and turquoise module genes have revealed the remarkable roles of these molecules during the immune response and, specifically, in the myeloid cell activation, leukocyte activation, oxygen carrier activity, replication fork processing, and bicarbonate transport. Besides, we have analyzed a recently published data set of scRNA-seq related to PBMCs of MS. We have used Louvain clustering and identified the marker for the 12 cell populations. Our results have indicated that *HBD*, *SLC25A37*, *HBM*, *SELENBP1*, and *SLC4A1* are the remarkably up-regulated genes in the PBMCs of MS patients. Then, we validated the expression level of *HBD* as a critical gene introduced in mitochondrial stress of white blood cells in the healthy donors, treatment-naive MS patients, and treated MS patients. In the following, the critical roles of *HBD* and *SLC25A37* in oxidative stress based on the available literature are discussed.

For the *HBD* gene expression analysis in the individual cells (both MS and control), we performed a cluster comparison of *HBD* gene expression between the control and MS samples. It appears that Plasmacytoid DC and γδ T cells from MS patients have higher expression of the *HBD* gene, but DC, Monocytes, Naive B cells, and Treg cells show lower expression.

The increased expression of hemoglobin, e.g., HBD, has been reported in inflammatory conditions. Kobayash et al. have shown that the expression of *HBD* has been considerably increased in patients with vasculitis (29). Moreover, Brunyanszki et al. have indicated that critical inflammatory illness, e.g., severe burn injury and sepsis, can result in the up-regulation of *HBD* in the PBMCs of the affected patients. Besides, HBD expression has been associated with decreased H_2_O_2_-induced damage to the nuclear and the mitochondrial DNA of PBMCs of patients with sepsis (30). Consistent with this, the up-regulation of *HBD* in the leukocytes of patients with sepsis has been attributed to pro-inflammatory conditions, in which HBD can serve as a cytoprotective factor (31). In line with these findings, Särkijärvi et al. have shown that the gene expression of hemoglobin-β and hemoglobin-α2 are both up-regulated in the mononuclear blood cells of the monozygotic MS patients, indicating the potential role of hemoglobin in the pathogenicity of MS (32). Appealingly, increased hemoglobin expression has been documented in the pyramidal neural cells of MS patients (33–35). Consistent with these, the expression level of HBD in treatment-naïve MS patients has been considerably elevated compared to healthy individuals. Of interest, following immunomodulatory therapies, i.e., fingolimod, DMF, and IFNβ-1α, and attenuation of inflammation, HBD expression level in the PBMCs from studied patients has been remarkably reduced. There are two theories for the hemoglobin up-regulation in inflammatory conditions; one school of thought advocates the protective role of hemoglobin against oxidative stress in inflammation (30, 31), and the other one states that the increased HBD expression might be stemmed from the increased differentiation and mitosis of hematopoietic stem cells to give rise the immune cells. Lotan et al. have indicated that pleocytosis is associated with a worse prognosis in MS patients; their results have demonstrated that pleocytosis is associated with inferior annualized relapse rate and the expanded disability status scale score in RRMS patients (36). In line with this, Brunyanszki et al. have shown increased expression of hematopoietic stem cell markers in the PBMCs of patients who have experienced burn injury (30). Additionally, it has been shown that the protein expression of hemoglobin is up-regulated during monocyte differentiation, and its level is decreased as the differentiation progresses (37).

*SLC25A37* is responsible for regulating iron homeostasis *via* transferring iron into the mitochondria matrix. Recent findings indicate that siRNA-mediated mitoferrin-1 silencing can substantially decrease the level of ROS (38). In line with this, the knockdown of mitoferrin 1 has been associated with decreased ROS production in Alzheimer’s disease (39). Indeed, ROS overproduction and mitochondrial damage are among the main culprits of MS pathogenicity both in immune cells and oligodendrocytes (6). Consistent with these findings, Gonzalo et al. have demonstrated that the mitochondrial respiratory chain complexes are remarkably damaged. The ROS production is considerably increased in MS patients compared to control individuals (9). Our bioinformatic analysis displayed *SLC25A37* as one of the up-regulated genes in the PBMCs of MS patients. However, further investigations are required to clarify these conflicting results and shed new light on disease pathophysiology.

Besides, our analysis has revealed that *HBM*, *SELENBP1*, and *SLC4A1* are also up-regulated in the PBMCs of MS patients; however, further investigations are needed to be conducted to investigate whether they have roles in the oxidative stress of MS or not.

Our study has several strengths. First, it is the first study that integrated the data from bioinformatics and *ex vivo* study to present the role of *HBD* in the pathogenesis of MS. Second, it is the first study investigating the effect of fingolimod, DMF, IFNβ-1α, and GA on the expression level of HBD in MS patients. However, the current study has several limitations as well. First, we could not measure the protein expression of HBD. Second, we did not do the age matching between the age of the patients and healthy donors.

## Conclusion

The current study has indicated that *HBD* expression in PBMCs from MS patients is substantially up-regulated and can be considerably down-regulated by the immunomodulatory therapies, i.e., fingolimod, DMF, and IFNβ-1α. The increased expression of HBD in the PBMCs of MS patients can be stemmed from the protective role of hemoglobin against oxidative injury and the inflammatory nature of MS, which can lead to increase differentiation and mitosis of hematopoietic stem cells. This study provides a novel insight into the role of mitochondrial oxidative stress in MS pathogenicity. It offers an opportunity for further investigations regarding the role of HBD in MS pathogenicity.

## Data Availability Statement

The original contributions presented in the study are included in the article/**Supplementary Material**. Further inquiries can be directed to the corresponding authors.

## Ethics Statement

The studies involving human participants were reviewed and approved by Ethics Committee of Tabriz University of Medical Sciences (IR.TBZMED.REC.1399.074). The patients/participants provided their written informed consent to participate in this study.

## Author Contributions

Conceptualization, AD. Writing, AD. Data curation, HS, MA, and NH. Formal analysis, AD, PL, ZA, and MP. Funding, VR. Supervision, BB and VR. All authors contributed to the article and approved the submitted version.

## Conflict of Interest

The authors declare that the research was conducted in the absence of any commercial or financial relationships that could be construed as a potential conflict of interest.

## Publisher’s Note

All claims expressed in this article are solely those of the authors and do not necessarily represent those of their affiliated organizations, or those of the publisher, the editors and the reviewers. Any product that may be evaluated in this article, or claim that may be made by its manufacturer, is not guaranteed or endorsed by the publisher.

## Abbreviations

MS, Multiple sclerosis; DEGs, differentially expressed genes; scRNA-seq, single-cell RNA sequencing; RRMS, relapsing-remitting multiple sclerosis; DMF, dimethyl fumarate; IFNβ-1α, interferon-beta 1-alpha; CNS, central nervous system; ROS, reactive oxygen species; PBMCs, peripheral blood mononuclear cells; WGCNA, Weighted gene co-expression network analysis; GEO, Gene Expression Omnibus; TOM, topological overlap matrix; dis-TOM, dissimilarity TOM; ME, module eigengene; GS, gene significance; MM, module membership; GO, Gene Ontology; MDOCE, Molecular Complex Detection; CSF, Cerebrospinal fluid; PCA, principal component analysis; GAPDH, Glyceraldehyde-3-phosphate dehydrogenase; HBD, Hemoglobin Subunit Delta; HBM, Hemoglobin Subunit Mu; SLC4A1, Solute Carrier Family 4 Member 1; LILRA5, Leukocyte Immunoglobulin Like Receptor A5; SLC25A37, Solute Carrier Family 25 Member 37; SELENBP1, Selenium Binding Protein 1; ALYREF, Aly/REF Export Factor; SNRNP40, Small Nuclear Ribonucleoprotein U5 Subunit 40; HINT3, Histidine Triad Nucleotide Binding Protein 3; STRING, Search Tool for the Retrieval of Interacting Genes/Proteins; AHSP, Alpha Hemoglobin Stabilizing Protein; HINT3, Histidine Triad Nucleotide Binding Protein 3; DNAJC14, DnaJ Heat Shock Protein Family, Hsp40) Member C14; STRADB, STE20 Related Adaptor Beta; MAT2A, Methionine Adenosyltransferase 2A; GLUD2, Glutamate Dehydrogenase 2; ATAD2B, ATPase Family AAA Domain Containing 2B; GLUD1, Glutamate Dehydrogenase 1.
