# Supplementary material for: The Role of Hemoglobin Subunit Delta in the Immunopathy of Multiple Sclerosis: Mitochondria Matters
Source: Front Immunol. 2021 Aug 24;12:709173. doi: 10.3389/fimmu.2021.709173 (PMC8421544; doi:10.3389/fimmu.2021.709173)

**Supplementary legends (Table and figures):**

**Table S1:**

**Table S1:** Module color characteristics of GSE138266. The co-expression modules were identified by WGCNA. The Grey module was failed to classify as a distinct co-expression module, and this module was eliminated from further analysis. A p-value less than 0.05 is statistically significant.

**Figure S1:** Sample dendrogram and trait heatmap. The color is proportional to the disease status (Red: healthy samples and white: MS samples).

**Figure S2:** The determination of the soft threshold in the WGCNA algorithm. The approximate scale-free fit index can be attained at the soft-thresholding power of 8. The left panel shows the scale-free fit index (y-axis) as a function of the soft-thresholding power (x-axis). The right panel displays the mean connectivity (degree, y-axis) as a function of the soft-thresholding power (x-axis).

**Figure S3:** Cluster dendrogram and module assignment from WGCNA. The branches correspond to highly interconnected groups of genes. Colors in the horizontal bar represent the modules.

**Figure S4:** Module-trait relationship. Each row corresponds to a module eigengene and the column corresponds to MS patient’s status. The numbers in each cell represent the corresponding correlation and p-value.

**Figure S5:** Module features of gene significance (GS) and module membership (MM). Modules significantly correlated with status (control vs. patient). Selection of best hubgenes for reconstruction of co-expression network in turquoise module. Hubgenes with GS and MM > 0.86.

**Figure S6:** Calculated marker genes using Scanpy pipeline for each cluster

**Table S1.**

| **Module colure** | **Correlation** | **P-value** | **Genes number** |
| --- | --- | --- | --- |
| Turquoise | 0.93 | 2.00E-12 | 898 |
| Yellow | -0.79 | 8.00E-07 | 295 |
| Black | -0.75 | 7.00E-06 | 190 |
| Salmon | 0.74 | 9.00E-06 | 125 |
| Tan | 0.61 | 7.00E-04 | 136 |
| Greenyellow | 0.48 | 0.01 | 157 |
| White | -0.42 | 0.03 | 48 |
| Red | 0.43 | 0.03 | 229 |
| Paleturquoise | 0.38 | 0.05 | 45 |
| Orange | 0.31 | 0.1 | 60 |
| Darkred | -0.29 | 0.1 | 86 |
| Royalblue | -0.29 | 0.1 | 90 |
| Skyblue | 0.24 | 0.2 | 47 |
| Darkgrey | -0.26 | 0.2 | 64 |
| Darkturquoise | -0.26 | 0.2 | 69 |
| Darkgreen | 0.26 | 0.2 | 79 |
| Lightyellow | -0.25 | 0.2 | 99 |
| Lightgreen | -0.25 | 0.2 | 102 |
| Grey60 | -0.25 | 0.2 | 110 |
| Lightcyan | 0.24 | 0.2 | 111 |
| Midnightblue | -0.27 | 0.2 | 112 |
| Purple | -0.25 | 0.2 | 166 |
| Pink | -0.25 | 0.2 | 185 |
| Green | -0.27 | 0.2 | 246 |
| Brown | 0.24 | 0.2 | 312 |
| Violet | -0.22 | 0.3 | 45 |
| Steelblue | 0.21 | 0.3 | 46 |
| Cyan | 0.2 | 0.3 | 114 |
| Magenta | 0.2 | 0.3 | 168 |
| Darkolivegreen | 0.17 | 0.4 | 42 |
| Darkorange | 0.16 | 0.4 | 51 |
| Grey | 0.16 | 0.4 | 77 |
| Saddlebrown | 0.13 | 0.5 | 46 |
| Blue | 0.087 | 0.7 | 350 |

**Figure S1:**

**
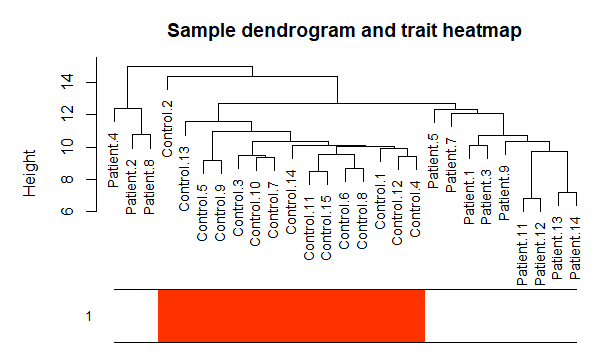
**

**Figure S2:**

**
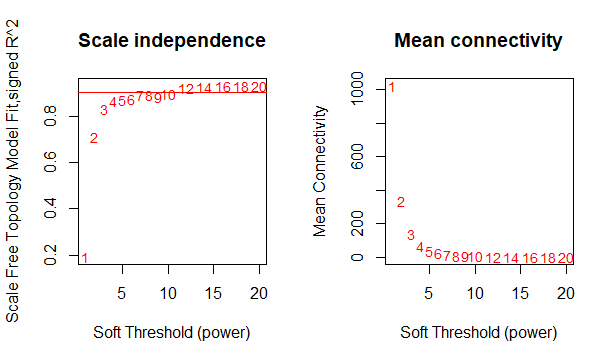
**

**Figure S3:**

**
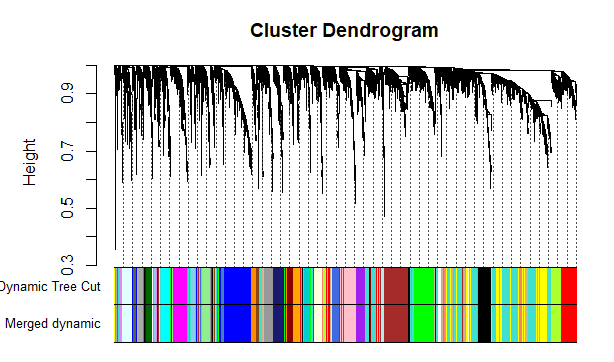
**

**Figure S4:**

**
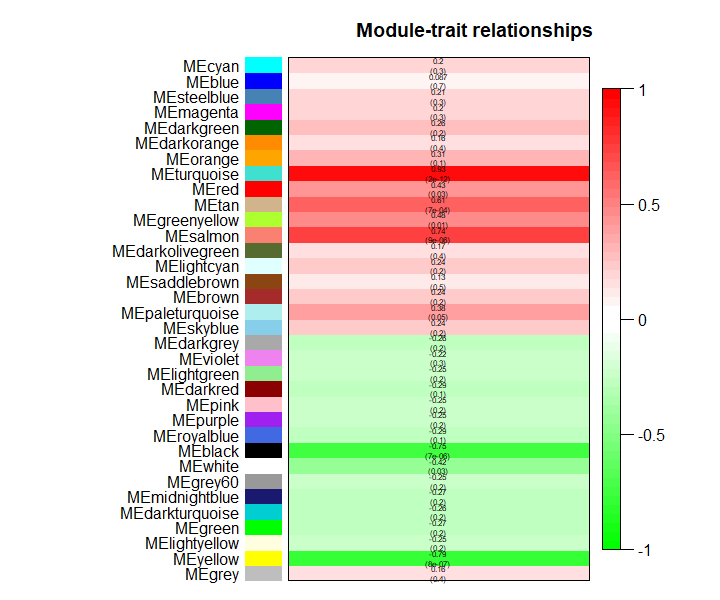
**

**Figure S5:**

**
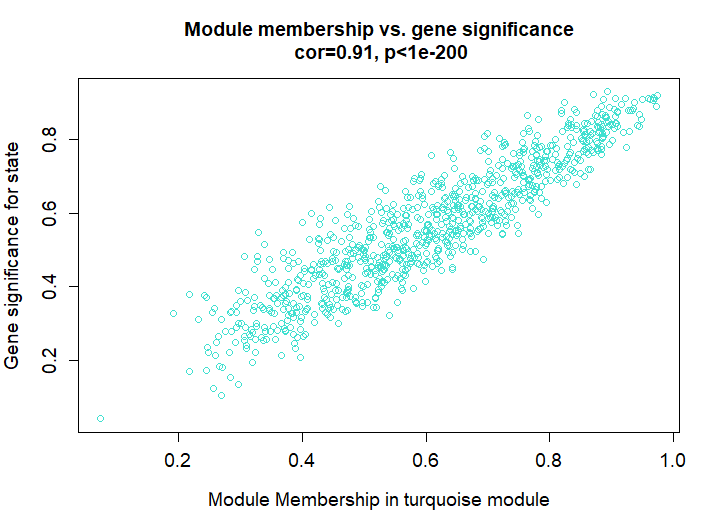
**

**Figure S6:**


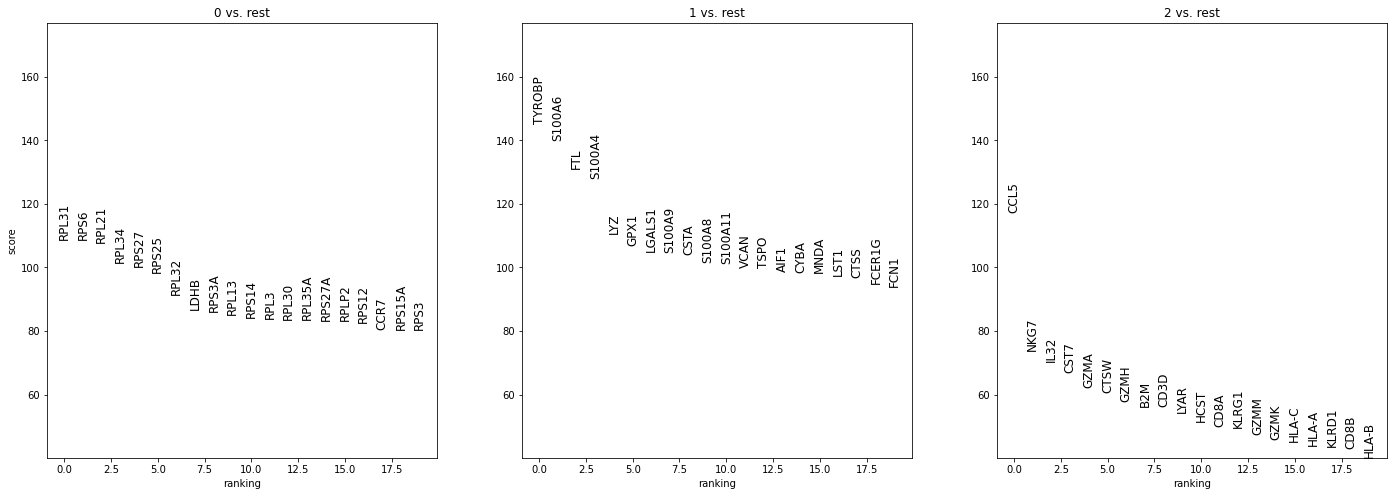

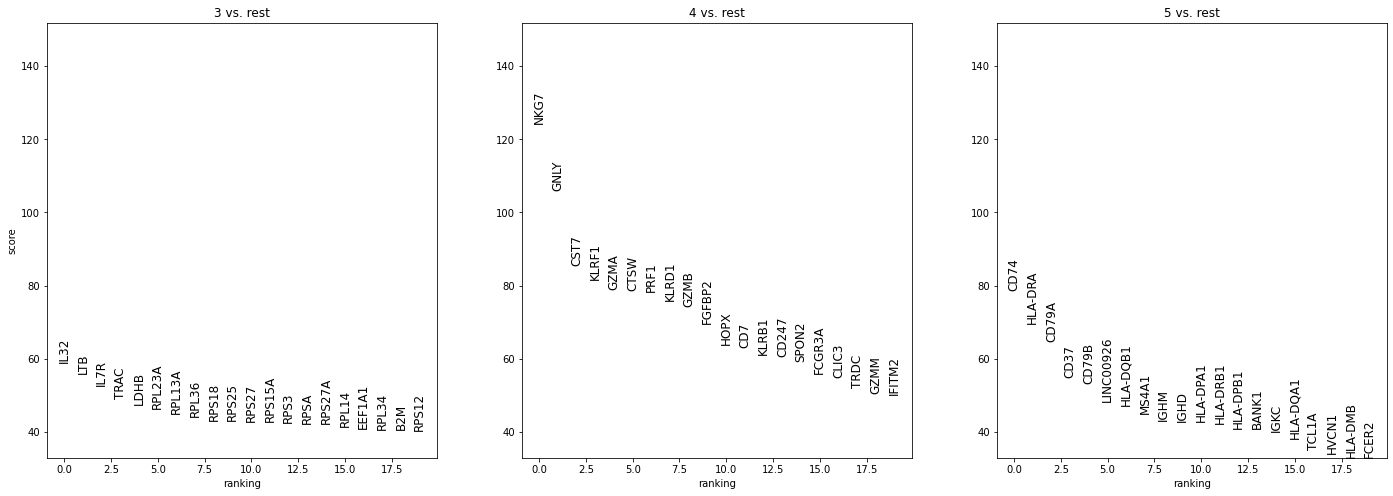

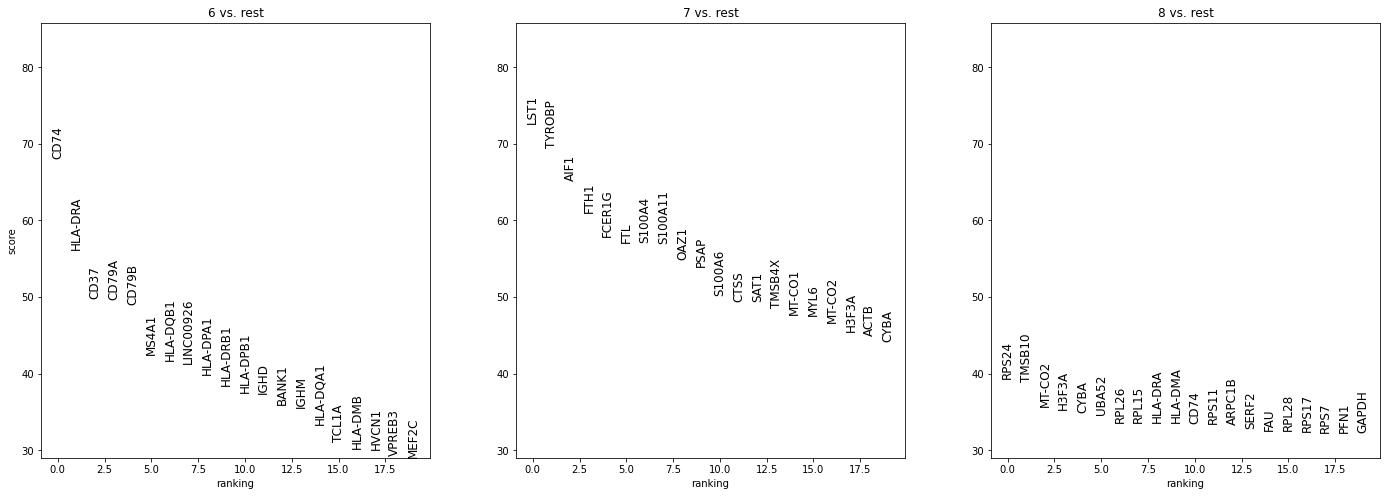

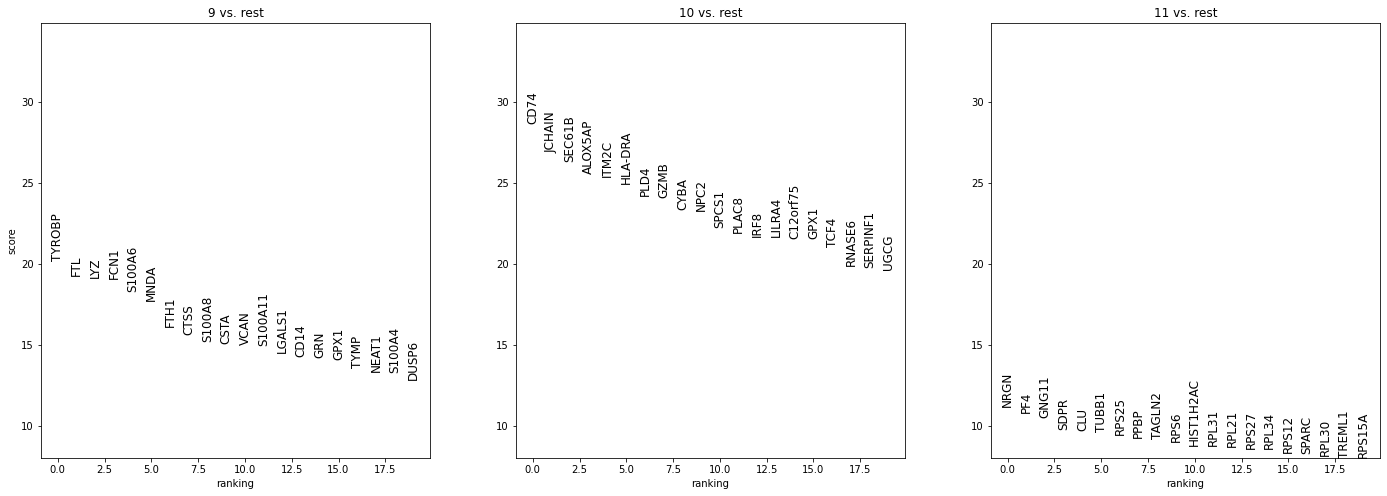

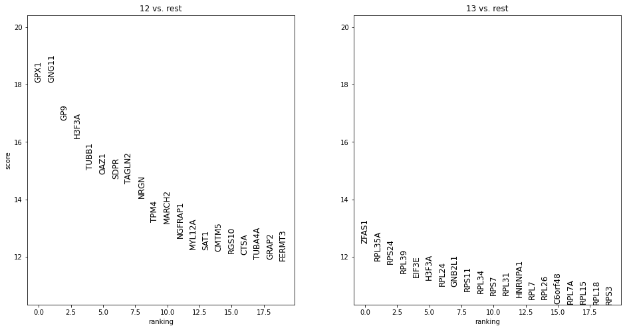

Supplement: Supplementary file 1 [file DataSheet_1.docx]
